# A rare case of a 34-year-old patient diagnosed late with Kallmann syndrome: case report

**DOI:** 10.11604/pamj.2022.43.67.36802

**Published:** 2022-10-10

**Authors:** Eppy Buchori Aristiady, Desiree Alberta

**Affiliations:** 1 Department of Radiology, Dr Hasan Sadikin Hospital, Faculty of Medicine, Universitas Padjadjaran Jl. Sederhana no. 39, Pasteur, Sukajadi, Bandung, Jawa Barat, 40161, Indonesia

**Keywords:** Kallmann syndrome, hypogonadotropic-hypogonadism, anosmia, olfactory bulb, case report

## Abstract

Kallmann syndrome (KS) is a rare genetic disorder manifested by the combination of hypogonadotropic-hypogonadism and olfactory dysfunction. It is usually diagnosed at 14 - 16 years of age due to delayed puberty. However, delays in diagnosis have been reported in a few cases. We presented a 34-year-old man presented with primary infertility as the chief complaint. Physical examination revealed bilateral gynecomastia, Tanner stage 2, and anosmia. Hormonal studies show a hypogonadotropic hypogonadism profile. Genetic testing revealed a normal male karyotype. Abdominal ultrasonography (USG) revealed a small prostate, and testicular USG demonstrated small testicles. Neuroimaging study revealed olfactory bulb agenesis and hypoplasia of the olfactory sulcus. Treatment was done by testosterone replacement therapy, and the patient is now on a regular follow-up. In conclusion, suspected clinical features of KS may guide the diagnosis with comprehensive hormonal and imaging studies. However, the reported patient was diagnosed extremely late.

## Introduction

Kallmann syndrome (KS) is a hypogonadotropic-hypogonadism disorder associated with olfactory disorders such as hyposmia or anosmia [1]. It is now known as olfactogenital dysplasia to assert the association between olfactory bulb agenesis and hypogonadism. This congenital condition is a rare genetic hormonal disorder, with an incidence estimated between 1: 8,000 and 1: 10,000 in men. Prevalence in women is rare, with a male to female ratio of 5: 1 [1,2]. Hypogonadotropic hypogonadism and hyposmia are the main clinical features of KS. Hypogonadotropic hypogonadism is characterized by low testosterone, luteinizing hormone (LH), and follicle-stimulating hormone (FSH) serum levels. Other less common features are labiopalatoschisis, hearing loss, color blindness, short fourth metacarpal, cardiovascular anomalies, osteoporosis, and urogenital problems, such as unilateral renal agenesis and cryptorchidism [3]. Diagnosis of KS can be guided by clinical manifestations and confirmed by hormonal profiles. In most cases, a brain's magnetic resonance imaging (MRI) scan may visualize a lack of olfactory bulb and sulcus [4]. As this disorder is relatively rare, late diagnosis is possible. As a consequence, the outcome of the treatment might not be optimal. Herein, we present a case of KS lately diagnosed in a 34-year-old Indonesian male patient.

## Patient and observation

**Patient information:** a 34-year-old Indonesian male presented at Dr. Hasan Sadikin General Hospital Bandung with primary infertility as the chief complaint. He had ever conducted a pregnancy program with his wife. Due to the abnormal hormonal assay results, he planned to do further examinations. He was circumcised. Morning erections were still notable. He did not complain of any disturbances in sexual activity and ejaculation. He did not realize any abnormalities since he was a teenager.

He had never smelled anything since he was a child. A family history of anosmia in the family is not found. He admitted that his voice was high-pitched. He had no history of seizures, blurring of vision, color blindness, and hearing loss. He had normal developmental milestones and no intellectual disturbances. He has siblings, and a similar complaint is not reported.

**Figure 1:**
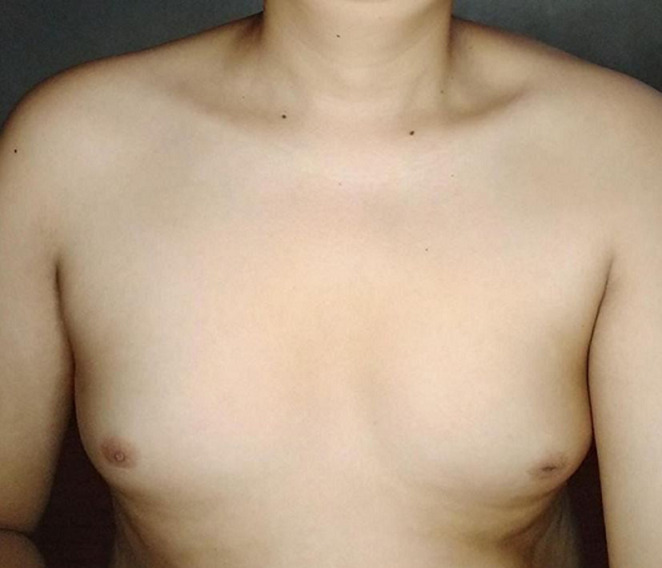
bilateral gynecomastia

**Figure 2:**
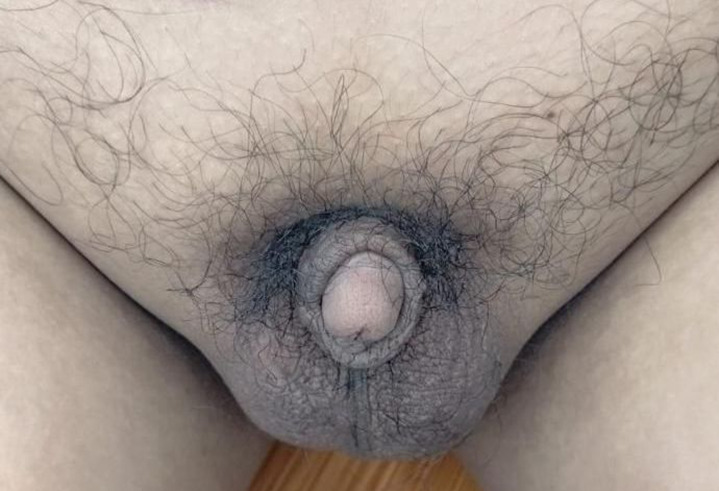
micropenis with bilateral descended prepubertal testes and sparse pubic hair

**Clinical findings:** physical examination showed an adult man profile. His body weight, height, and body mass index are 67kg, 160cm, and 26kg/m´, respectively. He had no galactorrhea, but bilateral gynecomastia was noted (Figure 1). His secondary sexual characteristics were in Tanner´s stage 2. He showed no hair over the face and sparse hair over the axillae and pubic area. Both testes were still palpated in the scrotum, measuring 2 ml using the orchidometer, with a penile length of 2.0 cm, and it is considered a microphallus (Figure 2). Neurologic examination was normal, except for the decreased sense of smell.

**Figure 3:**
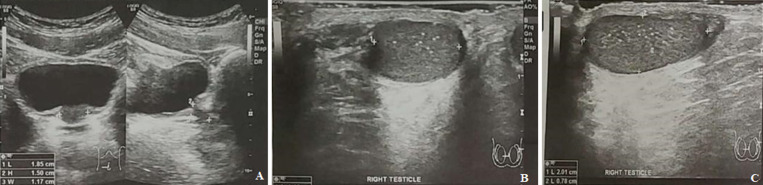
ultrasonography images showing (A) small prostate (arrow); (B and C) small testes with microcalcification within them (R: right, L: left) (arrow)

**Figure 4:**
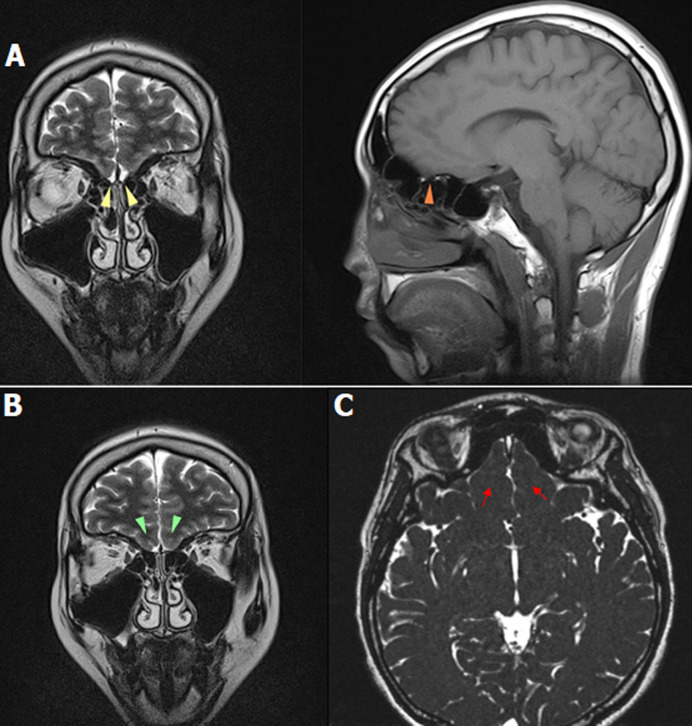
a coronal and sagittal T2-weighted MRI image showing bilateral olfactory bulbs agenesis (A), hypoplastic right olfactory sulcus, and aplastic left olfactory sulcus (B), an axial constructive interference in steady state (CISS) image showing an abnormal olfactory sulcus in an axial plane of CISS (C)

**Figure 5:**
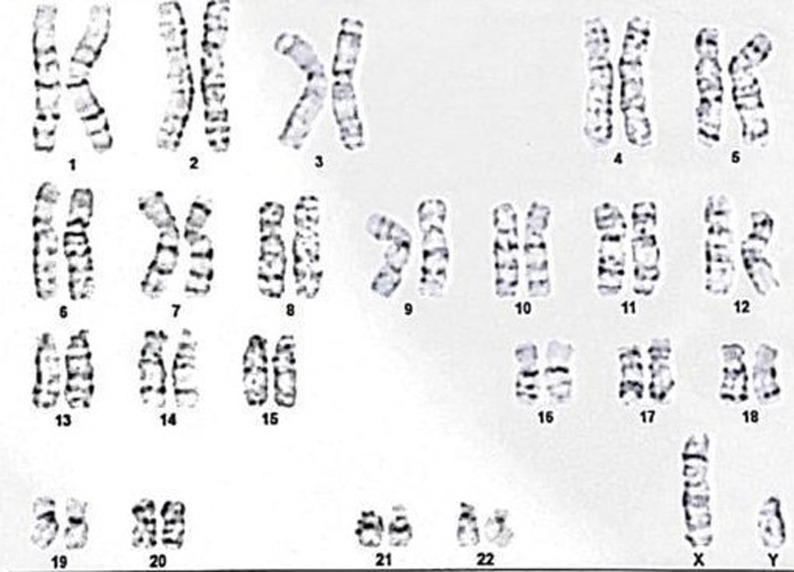
karyotyping analysis shows a 46XY pattern

**Diagnostic assessment:** hormonal assays described a hypogonadotropic hypogonadism profile. It revealed total testosterone levels of 5.58 ng/dL (normal value 249-836 ng/dL), LH levels of <0.5 IU/ mL (normal range 0.57-12.07), and FSH levels of 1.83 IU/mL (normal value 0.95-11.95). The prolactin was within the normal limit with a serum level of 4.76 ng/mL (normal value 3.46-19.4 ng/mL). Abdominal ultrasonography revealed a normal bladder with small prostate (Figure 3). The findings of scrotal sonography were small testes with a high position in the scrotum. The volume of the right and left testes was 1 cc and 1.2 cc, respectively. Microcalcification was found in both tests. Doppler examination showed normal vascularization of the testes. Brain MRI findings of this patient revealed an absent olfactory bulb and narrowed olfactory sulcus, which is consistent with Kallmann syndrome (Figure 4). Genetic profiles were determined in this patient. Karyotyping showed a 46XY pattern (Figure 5). Underdeveloped external genitalia, high position of testes, gonadal dysgenesis, and micropenis were the most likely syndrome. These findings are consistent with the feature of hypogonadism. Furthermore, a mutation in the CHH gene is suspected. Evaluation of other genes of sex development disorder is still required.

**Therapeutic interventions:** the patient was given testosterone replacement therapy to induce virilization. An oral testosterone undecanoate regimen was consumed in a dosage of 40 mg daily for two weeks. Follow-up will be regularly conducted by evaluating the clinical features and hormonal tests.

**Follow-up and outcome:** the patient was evaluated after receiving therapy for two weeks. No significant increment of penile length, testis size, and pubic hair thickness. The size was 2.1 cm, 2.5 ml using an orchidometer, and similar pubic hair thickness, respectively. Treatment was discontinued due to the patient´s economic issue.

**Patient perspective:** the patient was pleased with the care he received throughout therapy. He hoped he would restore fertility.

**Informed consent:** written informed consent was obtained from the patient for participation in our study.

## Discussion

We report a case of KS in a man diagnosed late in his age. KS combines an isolated form of hypogonadotropic hypogonadism and a lack of sense of smell. These features are due to defects in olfactory neurons, including olfactory tracts and bulbs, and failed migration of GnRH-secreting neurons to the hypothalamus regions, specifically in the preoptic area. As a result, secondary testicular failure occurs. Genetic mutations are the base of the neurological pathological condition [3].

KS can be diagnosed in neonates with micropenis and cryptorchidism. It is mostly diagnosed in adolescents due to the absence of secondary sexual development [3]. The current study reported the patient´s clinical presentation with gynecomastia and micropenis. Our case showed small testes, consistent with the KS feature, during the venereological examination. However, the patient is diagnosed extremely late in the present case due to the unawareness of his low stage of secondary sexual development. Culture and taboo might contribute to the late diagnosis, whereas clues of anosmia and lack of signs of puberty are discovered by patients. A similar report was presented by Arkoncel and associations [4]. Individuals with KS may present other abnormalities, including color blindness, cleft palate, high-arched palate, sensorineural hearing loss, cardiovascular anomalies, and renal agenesis [3]. However, the current case did not demonstrate any of these abnormalities. Arkoncel *et al*. reported KS patients with other facial anomalies, smooth philtrum, and congenital absence of puncta, which are uncommonly seen in KS [5]. Decreased sexual desire, erectile dysfunction, and decreased muscle strength may present in male patients with KS. Females usually present with amenorrhea or dyspareunia [6]. Our case showed a normal presentation of these features.

Small testes and micropenis are commonly found in KS, as in our patient [5]. Ultrasonography findings showed small testes consistent with the hypogonadotropic pattern in KS. Low testosterone and LH serum levels confirm this gonadotropin deficiency. Cryptorchidism, remnant ectopic testicular tissues, such as in the abdomen, should be confirmed and treated first before hormonal replacement therapy. Testicular secretions evaluation is useful to ensure the presence of cryptorchidism and assess Leydig cells' response to human chorionic gonadotropin [6]. KS is mainly transmitted in an X-linked form. Other less common forms of inheritance include autosomal dominant and recessive patterns. Therefore, it is also known as a heterogeneous genetic disorder [7]. CHH gene was suspected to be mutated in the reported patient. However, other gene mutations in de novo or inherited patterns still can not be ruled out. Limited genetic karyotyping examination becomes the obstacle in this report.

MRI is the first recommended imaging in suspected KS cases. It is superior in visualizing the morphological abnormalities of the olfactory apparatus. Furthermore, it is precise in detecting even very little damage in this area. The accuracy in measuring the volume of the olfactory bulb in various pathological conditions raises its strength [8]. Olfactory bulbs are located along with the cribriform plate. A sulcus found between the gyrus rectus and the medial orbital gyrus is the olfactory sulcus. Patients with suggestive clinical findings of KS should undergo radiological examination. A coronal view may optimally show the hypoplasia or aplasia of olfactory bulbs and tracts, which are the most common findings in KS. Complete anosmia in the present case is believed to be due to the absence of the olfactory bulbs [8]. Visualization of the olfactory sulcus of the frontal lobes is preferred in the axial view. This sulcus may variably appear normal, hypoplastic, or aplastic. It can also be seen in the paramedian sagittal section, but its sensitivity is lower than the axial view. In addition, the high-resolution coronal T2- and T1-weighted images are recommended to evaluate the olfactory apparatus [8].

Testosterone replacement therapy is the main standard of treatment in KS. The main goal of this patient was to effectively develop fertility. Besides, it also induces the development of secondary sexual characteristics. Immediate treatment is to restore metabolic and bone activity and improve the psychosocial effects in patients with KS [9]. Testosterone is usually preferred to pulsatile GnRH treatment because of its lower cost and availability, especially in a low-resource setting. Clinical response to the therapy should be followed up regularly as future fertility becomes significant attention [10]. However, treatment was not continued after two weeks of receiving therapy due to the patient´s economic issue. The possibility of restoring the olfactory function is slight. No cure is available for this concern as the etiology of hyposmia is due to the pathological olfactory apparatus. A complete absence of olfactory structures means regaining olfaction is not possible. Even the likelihood of restoration in patients with hypoplastic olfactory bulbs and tracts is narrow [6]. Therefore, patient reassurance and education are necessary.

## Conclusion

Kallmann syndrome is a rare genetic hormonal disorder that is now called olfactogenital dysplasia, characterized by hypogonadotropic-hypogonadism and lack of olfactory function. KS should be suspected clinically in patients with one or both of these features because the late diagnosis of KS will impact the outcome after treatment. Late diagnosis is demonstrated in this case. The prognosis of patients with KS diagnosed early is better than late.

## References

[1] Dzemaili S, Tiemensma J, Quinton R, Pitteloud N, Morin D, Dwyer AA (2017). Beyond hormone replacement: quality of life in women with congenital hypogonadotropic hypogonadism. Endocr Connect.

[2] Ribeiro RS, Abucham J (2008). [Kallmann syndrome: a historical [corrected] clinical and molecular review]. Arq Bras Endocrinol Metabol.

[3] Boehm U, Bouloux PM, Dattani MT, de Roux N, Dodé C, Dunkel L (2015). Expert consensus document: European Consensus Statement on congenital hypogonadotropic hypogonadism--pathogenesis, diagnosis and treatment. Nat Rev Endocrinol.

[4] Zhang Z, Sun X, Wang C, Wang G, Zhao B (2016). Magnetic Resonance Imaging Findings in Kallmann Syndrome: 14 Cases and Review of the Literature. J Comput Assist Tomogr.

[5] Arkoncel ML, Arkoncel FR, Lantion-Ang FL (2011). A case of Kallmann syndrome. BMJ Case Rep.

[6] Dash PK, Raj DH (2014). Biochemical and MRI findings of Kallmann´s syndrome. BMJ Case Rep.

[7] Mitchell AL, Dwyer A, Pitteloud N, Quinton R (2011). Genetic basis and variable phenotypic expression of Kallmann syndrome: towards a unifying theory. Trends Endocrinol Metab.

[8] Zaghouani H, Slim I, Zina NB, Mallat N, Tajouri H, Kraiem C (2013). Kallmann syndrome: MRI findings. Indian J Endocrinol Metab.

[9] Ach T, Marmouch H, Elguiche D, Achour A, Marzouk H, Sayadi H (2018). A case of Kallmann syndrome associated with a non-functional pituitary microadenoma. Endocrinol Diabetes Metab Case Rep.

[10] Kim SH (2015). Congenital Hypogonadotropic Hypogonadism and Kallmann Syndrome: Past, Present, and Future. Endocrinol Metab (Seoul).

